# Can Be miR-126-3p a Biomarker of Premature Aging? An Ex Vivo and In Vitro Study in Fabry Disease

**DOI:** 10.3390/cells10020356

**Published:** 2021-02-09

**Authors:** Alessia Lo Curto, Simona Taverna, Maria Assunta Costa, Rosa Passantino, Giuseppa Augello, Giorgia Adamo, Anna Aiello, Paolo Colomba, Carmela Zizzo, Marco Zora, Giulia Accardi, Giuseppina Candore, Daniele Francofonte, Tiziana Di Chiara, Riccardo Alessandro, Calogero Caruso, Giovanni Duro, Giuseppe Cammarata

**Affiliations:** 1 Institute for Research and Biomedical Innovation (IRIB), National Research Council (CNR), 90146 Palermo, Italy; alessia.l_86@hotmail.it (A.L.C.); simona.taverna@irib.cnr.it (S.T.); giuseppa.augello@irib.cnr.it (G.A.); giorgia.adamo@irib.cnr.it (G.A.); paolo.colomba@irib.cnr.it (P.C.); carmela.zizzo@irib.cnr.it (C.Z.); marco.zora@irib.cnr.it (M.Z.); daniele.francofonte@irib.cnr.it (D.F.); riccardo.alessandro@unipa.it (R.A.); giovanni.duro@irib.cnr.it (G.D.); 2 Institute of Byophysics, National Research Council (CNR), 90146 Palermo, Italy; mariaassunta.costa@cnr.it (M.A.C.); rosa.passantino@cnr.it (R.P.); 3 Laboratory of Immunopathology and Immunosenescence, Department of Biomedicine, Neuroscience and Advanced Diagnostics, University of Palermo, 90134 Palermo, Italy; anna.aiello@unipa.it (A.A.); giulia.accardi@unipa.it (G.A.); giuseppina.candore@unipa.it (G.C.); calogero.caruso@unipa.it (C.C.); 4 Department PROMISE, School of Medicine, University of Palermo, 90127 Palermo, Italy; tiziana.dichiara@unipa.it; 5 Department of Biomedicine, Neuroscience and Advanced Diagnostics-Section of Biology and Genetics, University of Palermo, 90127 Palermo, Italy

**Keywords:** Fabry disease, microRNA, miR-126-3p, small extracellular vesicles, aging, senescence, endothelial cells, HUVEC

## Abstract

Fabry disease (FD) is a lysosomal storage disorder (LSD) characterized by lysosomal accumulation of glycosphingolipids in a wide variety of cytotypes, including endothelial cells (ECs). FD patients experience a significantly reduced life expectancy compared to the general population; therefore, the association with a premature aging process would be plausible. To assess this hypothesis, miR-126-3p, a senescence-associated microRNA (SA-miRNAs), was considered as an aging biomarker. The levels of miR-126-3p contained in small extracellular vesicles (sEVs), with about 130 nm of diameter, were measured in FD patients and healthy subjects divided into age classes, in vitro, in human umbilical vein endothelial cells (HUVECs) “young” and undergoing replicative senescence, through a quantitative polymerase chain reaction (qPCR) approach. We confirmed that, in vivo, circulating miR-126 levels physiologically increase with age. In vitro, miR-126 augments in HUVECs underwent replicative senescence. We observed that FD patients are characterized by higher miR-126-3p levels in sEVs, compared to age-matched healthy subjects. We also explored, in vitro, the effect on ECs of glycosphingolipids that are typically accumulated in FD patients. We observed that FD storage substances induced in HUVECs premature senescence and increased of miR-126-3p levels. This study reinforces the hypothesis that FD may aggravate the normal aging process.

## 1. Introduction

The syndromes of accelerated aging have been proposed as models to simplify the analysis of the aging process, by restricting the focus to a more definable area [1]. In humans, premature aging syndromes represent the conditions in which multiple tissues and organs show features of accelerated aging with early onset of deleterious changes, impairment of physiologic functions, and increased risk of disease and death [2,3,4]. Fabry disease (FD) is an inherited error of glycosphingolipid catabolism caused by an insufficient activity of α-galactosidase A [5], a progressive storage of intracellular globotriaosylceramide (Gb3) in vasculature, and the accumulation in extracellular space of lyso-Gb3 [6], a deacetylated soluble form of Gb3 [7]. In FD, the vascular complications are responsible for most symptoms, such as ischemic stroke, cardiac abnormalities, and progressive renal failure [8]. The cellular homeostasis alteration, caused by lysosomal engulfment, is the mechanism underlying FD, but other processes, such as inflammation and oxidative stress that affects mainly the vascular endothelium, are potentially involved in FD pathophysiology [9]. Nowadays, FD pathophysiology is not completely known. Recently, dysregulation of some aging hallmarks, such as telomere attrition and DNA damage, has been observed in FD patients, as compared to age-matched controls [10]. These data suggest that FD may be another disease associated with premature aging [11]. Cellular senescence is considered to be a cellular counterpart to aging of tissues and organisms. Senescence causes cell cycle arrest, leading to permanent proliferative block and loss of the cell’s capability to proliferate in response to growth factors or mitogens [12]. Although senescence plays a physiological role in development and maintenance of tissue homeostasis, this process is also a stress response triggered by insults associated with aging, such as oxidative stress, glycation, telomere shortening, side reactions, mutations, and aggregation of proteins [13]. Several recent evidences demonstrate that senescent cells are a source of circulating microRNAs (miRNAs) [14]. 

MiRNAs, are single-stranded and non-coding RNA molecules of 21–23 nucleotides that negatively regulate gene expression and are involved in broad spectrum of physiological and pathological conditions [15,16,17]. In addition to their intracellular functions, several evidences have demonstrated that some miRNAs are selectively packaged into cells derived extracellular vesicles (EVs) [18,19]. EVs contain a plethora of bioactive cargoes, including proteins, lipids, and nucleic acids; the lipid bilayer of EVs protects their contents from enzymatic degradation [20,21,22]. EVs have a key role in intercellular communication, regulating several cellular processes through autocrine and paracrine interactions [23,24]. The two major populations of EVs described are microvesicles and exosomes. Microvesicles are large membrane vesicles with their diameter size ranging between 150 to 1000 nm, budding directly from plasma membrane. Exosomes are small vesicles, ranging from 30 to 150 nm in diameter [25,26,27]. In this paper, we use the generic term of extracellular vesicles; considering the diameter size (about 130 nm), we indicate EVs as small extracellular vesicles (sEVs) [28]. MiRNAs contained in EVs, are able to influence the phenotype and biological functions of target cells [29,30]. Recently, it was also reported that EVs shuttled miRNAs associated with aging and aging-related disorders and can be useful as aging molecular markers [31,32]. Specific miRNAs, widely known as senescence-associated miRNAs (SA-miRNAs), regulate the expression of genes involved in pathways of longevity that act as cell cycle regulators [33]. MiR-126-3p, one of the most described SA-miRNAs, has a key role in cellular aging [30,34,35]. It is considered an endothelial cell-specific miRNA that governs vascular integrity and angiogenesis targeting specific mRNAs, such as C-X-C motif chemokine (CXCL12) [36], Vascular cell adhesion protein 1 (VCAM-1) [37], Sprouty-related EVH1 domain-containing protein1 (SPRED-1), and Phosphoinositide-3-Kinase Regulatory Subunit 2 (PIK3R2) [38,39]. In endothelial cells, miR-126-3p promotes angiogenesis by inhibiting endogenous VEGF repressors SPRED1 and PIK3R2 [40]. Several reports suggest that circulating miR-126-3p, released by ECs, exerts protective mechanism against vascular endothelial dysfunction [41]. Alterated plasmatic levels of miR-126-3p have been associated with vascular injury in several diseases, like diabetes [42], coronary artery disease, chronic kidney disease [43,44], and sepsis [45]. In FD patients, vascular impairment has been documented [46]. Moreover, it has been suggested that Gb3 accumulation in endothelium is able to dysregulate the activity of endothelial NO synthase (eNOS) [47]. Gb3 storage may alter caveolar stability and the downstream signal transduction of caveolar proteins, such as eNOS [48]. Moreover, eNOS deregulation, which reduces NO production, might increase the production of ROS [49]. In our previous work, we also found a significant alteration of miR-126-3p plasmatic levels in FD patients, compared to healthy subjects [50]. The aims of this study are to explore the hypothesis that FD patients could experience premature aging and to establish a potential link with FD pathogenic mechanism.

## 2. Materials and Methods

### 2.1. Patients Characteristics

A total of 90 subjects were recruited: 30 treatment-naïve Fabry patients and 60 healthy volunteers with normal enzyme activity and no mutations in GLA gene. The age and gender of patients were selected to be heterogeneous, while healthy control subjects with no history of cardiovascular disease were selected to provide an age range and gender distribution similar to the FD group (Table 1). The clinical diagnosis of FD was confirmed by mutation analysis in all patients. The patients were considered naïve if they harbor an FD causative mutation, according to Fabry Database (http://fabry-database.org), before starting the therapy. Affected patients showed classical symptoms of FD, including acroparasthesias, cornea verticillata, and angiokeratomas, in association with low or absent GLA activity and high levels of plasma lyso-Gb3 (Table 1). The study was carried out in accordance with the Declaration of Helsinki (2000) of the World Medical Association. The Ethical Committee of the University of Palermo approved the study protocol, and informed consent was obtained from all the subjects. Age, gender, and clinical data of the patients were reviewed and recorded (Table 1). 

### 2.2. Plasma Sample Acquisition and RNA Isolation

Blood samples were drawn, using an 18-Gauge needle, to avoid hemolysis, and collected into EDTA Tubes-Plasma (Cat# 367863). The samples were processed within one hour from the collection to minimize degradation. Plasma was extracted by firstly centrifugation of whole blood at 1900× *g*, for 10 min, at 4 °C, and successive centrifugation at 16,000× *g*, for 10 min, at 4 °C. All plasma samples were stored at −80 °C, until sEVs’ isolation and miRNA extraction, to minimize the effect of thawing. Total RNA was extracted and purified from plasma sEVs enriched fractions, using miRNeasy^®^ Mini kit (Qiagen, cat. No. 217004), according to standard protocol. RNA concentration was assessed, using RNA Nano 6000 Assay Kit of the Agilent Bio-analyzer 2100 System (Agilent Technologies, CA, USA). RNA quality was assessed with Eppendorf biophotometer D30. For this study, we used only RNA, with a ratio of A260/280 from 1.9 to 2.

### 2.3. GLA Gene Analysis

A genetic test of the α-Gal A gene was performed in all patients and controls (CTRs). DNA samples were extracted from whole blood, using a chromatography method (Gene lute Blood Genomic DNA Kit, Miniprep, Sigma-Aldrich, St. Louis, MO, USA), and, after the determination of concentrations by spectrophotometer, the amplification of GLA exons was performed. PCR products were purified and sequenced, using an automated DNA sequencer at Eurofins Genomics (Ebersberg, Germany).

### 2.4. sEVs’ Isolation from Plasma

In total, 2 mL of plasma was centrifuged at 1500× *g*, for 10 min, to eliminate cells; and at 3000× *g*, for 30 min, to discard dead cells and debris. The supernatant was centrifuged at 10,000× *g*, for 30 min, to eliminate the large EVs, and then used to purify the sEVs fraction by ultracentrifugation at 100,000× *g*, for 1 h 45min, at 4 °C, using a previously described procedure [50]. The final pellets containing the sEVs fractions were diluted in sterile PBS, for characterization, or stored at −80 °C.

### 2.5. Nanoparticle Tracking Analysis (NTA)

The sEVs’ size distribution analysis was performed by using a NanoSight LM10 (NanoSight Ltd., Minton Park UK). The particles of the samples were illuminated, using a laser light source, and the scattered light was captured by camera and analyzed, using Nanoparticle Tracking Analysis (NTA). NTA automatically tracked and sized particles according to Brownian motion and the diffusion coefficient (Dt). Results were displayed as a frequency size distribution graph and outputted to a spreadsheet.

### 2.6. TaqMan RT-qPCR miRNA Assays

The isolated miRNAs were retrotranscripted, using miScript Single Cell qPCR kit (Qiagen, Germany), according to the manufacturer’s protocol. The expression levels of miRNAs were evaluated with a SYBR green-based real-time quantitative PCR (RT-qPCR), using the Step one plus (Applied Biosystem, Waltham, MA, USA). For the amplification, we used miScript SYBR green PCR kit (Qiagen, Germany) according to the manufacturer’s protocol. The 20 µL PCR mixture included 2 µL reverse transcription product, 10 µL QuantiTect SYBR Green PCR Master Mix, 2 µL miScript Universal Primer, 2 µL miScript Primer Assay (specific for miR-126-3p, purchased from Qiagen, Hilden, Germany catalog No.-MS00003430), and 4 µL RNase-free water. The reaction mixtures were incubated at 95 °C for 15 min, followed by 40 amplification cycles of 94 °C for 15 s, 55 °C for 30 s, and 70 °C for 30 s. Triplicate samples and inter-assay controls were used. Therefore, for the normalization of RT-qPCR data, using the 2-ΔCT method, we used miR-30a for microRNAs extracted from EVs and RNU6b for microRNAs extracted from cells. Linear fold changes were calculated and plotted on scatter plots, using Prism (GraphPad Prism Software, San Diego, CA, USA).

### 2.7. Cell Culture and Treatments

Human umbilical vein endothelial cells (HUVEC ATCC^®^ CRL-1730™) were grown in Medium 200 (GIBCO) supplemented with hydrocortisone (1 µg/mL), human epidermal growth factor (10 ng/mL), basic fibroblast growth factor (3 ng/mL), heparin (10 µg/mL), gentamicin (10 µg/mL), amphotericin (0.25 µg/mL), and fetal bovine serum (2% *v/v*), at 37 °C, in a 5% CO_2_ atmosphere, at 95% humidity. For cell-replicative senescence, first passage cryopreserved HUVECs were grown and serially passaged until they reached senescence, as described previously. The number of population doublings (PDs) was calculated by using the following formula: PD = (ln [number of cells harvested]–ln [number of cells seeded])/ln2. For the experiments, cells were used at different cumulative population doublings (CPDs), that is, the sum of all PD. Cells studied in early passage (CPD < 20) were regarded as young cells, whereas those passaged more times were regarded as intermediate-age (CPD < 32) or senescent (CPD > 56) endothelial cells. For treatments, Globotriaosylsphingosine (Lyso-Gb3) and Globotriaosylceramide (Gb3) were prepared as described by Biancini et al., 2017, aliquoted, and stored at −20 °C. Before use, an aliquot of Gb3 or Lyso-Gb3 was added to the same amount of bovine serum albumin in phosphate buffered saline (PBS), and the resulting solution was diluted in culture medium. HUVECs were untreated or treated with 10 Gb3 or 25 nM Lyso-Gb3 for 24 h. Tert-butyl hydroperoxide (TBH), an oxidative stress inducer, was used at 60 µM concentration and kept in the same conditions. The optimum concentration of each reagent was selected according to the results of preliminary experiments performed to establish the cytotoxicity and the intracellular ROS induction of a large range of concentrations. 

### 2.8. Intracellular and HUVECs-Derived sEVs RNA Extraction 

HUVECs (2 × 10^6^), in the young, intermediate age, or senescence phase, were grown in 100 mm culture dish for 24 h, at 37 °C, in 5% CO_2_. Next, the medium was removed, and cells were washed twice with PBS. Culture medium was replaced with fresh medium supplemented with EV-depleted fetal bovine serum (GIBCO Exosome Depleted FBS), 10 µM Gb3, or 25 nM Lyso-Gb3, or 60 µM (TBH), and cells were cultured for 24 h. Next, conditioned medium (CM) was collected for exosome isolation, as above described. In brief, CM was centrifuged at 300× *g* for 10 min, then at 2000× *g* for 20 min, and finally at 10,000× *g* for 30 min. The supernatant was ultracentrifuged, using a SW 28 Swinging-Bucket Rotor (Beckman Coulter, Brea, CA, USA) at 110,000× *g*, for 70 min. The sEVs pellet was washed in phosphate-buffered saline (PBS), to eliminate contaminating proteins, and centrifuged again at 110,000× *g*, for 70 min. The final pellets containing the sEVs were re-suspended in PBS, and RNA extraction was carried out with miRNeasy^®^ Mini kit (Qiagen, cat. No. 217004), according to standard protocol. After removing the CM, adherent cells were washed twice with PBS and detached with trypsin-EDTA. Then, 1 × 10^6^ cells were collected and processed for intracellular RNA extraction with an miRNeasy^®^ Mini kit (Qiagen, cat. No. 217004), according to standard protocol.

### 2.9. Senescence-Associated β-Galactosidase Staining

HUVECs in young, intermediate age, or senescence phase were grown in a 4-well chamber slide (Nunc™ Lab-Tek™ II), at a density of 6 × 10^4^ cells/well, in 500 µL of culture medium, for 24 h. After treatments, HUVECs were fixed and stained for β -galactosidase activity, using a Senescence Cell Staining kit, following the manufacturer’s instructions (Sigma-Aldrich). The percentage of senescence-associate β-gal positive cells was determined by counting the number of blue cells within a sample, using a Zeiss Axioskop microscope (Carl Zeiss, Göttingen, Germany) with an X20 lens. Ten random fields were photographed for each passage, and the percentage of SA-β-gal-positive cells was calculated. RNA quality and concentration were assessed with RNA Nano 6000 Assay Kit of the Agilent Bioanalyzer 2100 System. For this study, we used RNA samples with RNA integrity number (RIN) > 7.5

### 2.10. Determination of Intracellular ROS Content by DCFH-DA assay

Intracellular ROS levels were evaluated by using 2′, 7′-Dichlorofluorescin diacetate (DCFH-DA), a cell-permeable ROS indicator (Wang and Josef, 1999). Inside cells, DCFH-DA is deacetylated by cellular esterases and oxidized to highly fluorescent 2′, 7′-Dichlorofluorescin (DCF) by intracellular ROS. HUVECs in the young phase were grown for 24 h, in a 96-well black plate, at a density of 104 cells/well, in 100 µL of phenol red-free culture medium, at 37 °C, in 5% CO_2_, and then treated or not with Gb3, Lyso-Gb3, or TBH for 24 h. Next, HUVECs were washed and incubated with 100 µM DCFH-DA in phenol red- and serum-free medium, for 1 h, at 37 °C, in 5% CO_2_, in the dark. After incubation, the cells were washed three times with PBS, and the fluorescence resulting from the production of intracellular ROS was measured in the Fluoroskan Ascent FL Thermo Scientific microplate reader (excitation and emission wavelengths at 485 and 528 nm, respectively). 

### 2.11. MTS Assay

Cell viability was evaluated by using CellTiter 96 Aqueous One Solution Cell Proliferation Assay (Promega, Madison, WI, USA), a colorimetric method for determining the number of viable cells in proliferation, according to manufacturer instructions.

### 2.12. Statistical Analyses

All calculations were made by using GraphPad Prism (GraphPad PrismSoftware, San Diego, CA, USA). Data from analysis of patients and controls were expressed as median ± standard deviation. Data from in vitro experiments were expressed as mean ± standard error of the mean from at least three independent experiments. Correlations between parameters were calculated by using Pearson correlation coefficient. Data in normal distribution and homogeneity of variance were analyzed by independent sample t-test between two groups. Comparison among multiple groups was analyzed by one-way analysis of variance (ANOVA), followed by Bonferroni’s correction. Statistical significance was assumed when *p* < 0.01.

## 3. Results

### 3.1. EVs miR-126-3p Levels in Healthy Age Classes 

**Figure 1:**
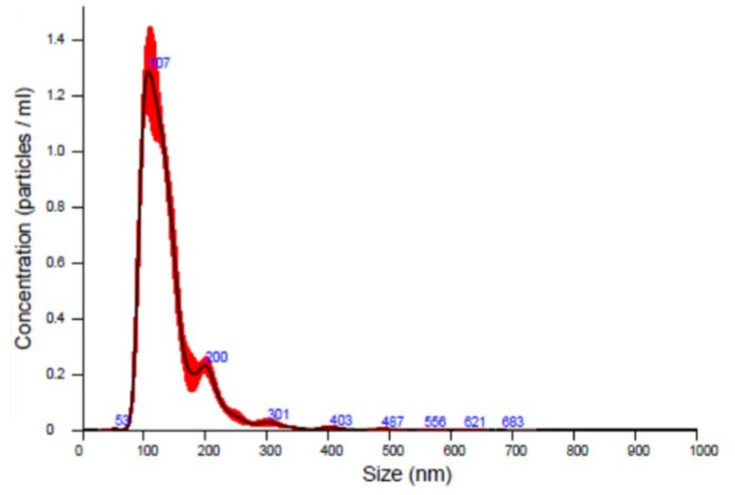
Representative Nanoparticle Tracking Analysis (NTA) analysis of small extracellular vesicles (sEVs) collected by ultracentrifugation method; sEVs isolated from plasma of different age classes of CTRs and FD EVs had a mean diameter of 138.9 +/− 1.9 nm and mode of 111.8 +/− 4.5 nm, here called small extracellular vesicles (sEVs).

**Figure 2:**
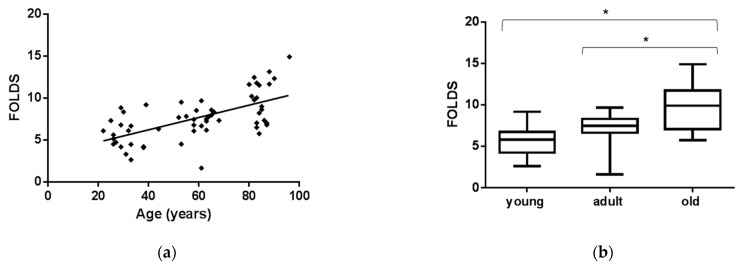
MiR-126-3p levels in sEVs of CTRs are modulated by aging. Scatterplots showing relative miR-126-3p expression according to age in CTRs (**a**). Box and Whiskers indicate median, minimum and maximum levels of miR-126-3p in sEVs from plasma of healthy CTRs divided in age class (24–44 years, *n* = 20), adult (53–68 years, *n* = 20) and old (81–96 years, n = 20) (**b**). CTs (cycle thresholds) resulting from qRT-PCR analysis were normalized with mir-30a; levels were calculated with 2-ΔCT method and expressed as folds, with respect to lowest value registered. Comparison among multiple groups was analyzed by one-way analysis of variance, followed by Bonferroni’s post hoc test. * *p* < 0.01.

EVs were collected from plasma of healthy subjects (CTRs) and FD patients by age group (Table 1). Briefly, EVs were isolated from 2 mL of plasma by ultracentrifugation, as described in the Methods section. EVs were characterized by nanoparticle tracking analysis (NTA). As shown in Figure 1, EVs collected had a diameter with mean of 139 +/− 2 nm and mode of 112 +/− 4 nm. These results demonstrated that differences of EVs’ average size of controls and FD patients, by age group, were not statistically significant. Considering the vesicles’ diameter-size observed, here we refer to this vesicle population as small extracellular vesicles (sEVs). In order to evaluate age-related changes, sEVs-miR-126-3p levels were measured in healthy subjects of different age classes. Plasma samples of 60 CTRs subjects were grouped into three age classes: young (24–44 years, n = 20), adult (53–68 years, n = 20), and old (81–96 years, n = 20). After RNA isolation, miR-126 levels were measured by RT-qPCR, and U6 small nuclear RNA (RNU6B) was used as endogenous control for normalizing miR-126-3p cycle threshold (CT) [51]. Since there is no unanimous consensus for the best control to normalize circulating miRNAs values, we also normalized the data by using CT of miR-30a, as previously described [50]. However, both normalization methods yielded similar results. We found that mean levels of miR-126-3p, carried in sEVs, isolated from 2 mL of plasma, increase progressively with age (correlation coefficient R = 0.411, *p* = 0.001) (Figure 2a), showing significant differences between the old and young groups, and the old group compared to adult. MiR-126-3p mean levels are expressed as folds, with respect to lowest value registered; in particular, we observed 5 ± 4, 5 ± 4 and 10 ± 5, respectively in young, adult and old group. ANOVA, followed by Bonferroni’s post hoc test, showed a *p*-value of *p* < 0.01 for young vs. old and adult vs. old (Figure 2b). This result confirmed that miR-126-3p contained in sEVs could be considered a premature aging biomarker.

### 3.2. sEVs miR-126-3p Levels in FD Patients of Different Ages

**Figure 3:**
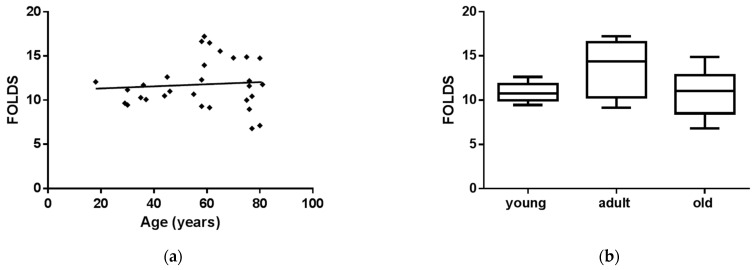
MiR-126-3p levels in sEVs of FD patients are not modulated by aging. Scatterplots showing relative miR-126-3p expression according to age in FD patients (**a**); miR-126-3p levels variation in sEVs of FD patients grouped in age class. Box and Whiskers indicate median, minimum, and maximum levels of miR-126-3p in sEVs from different age class of FD patients (30) (**b**). Data were calculated by qRT-PCR and represent mean ± SD of three different experiments analyzed in triplicate. CTs (cycle thresholds) resulting from qRT-PCR analysis were normalized with mir-30a; levels were calculated with 2-ΔCT method and expressed as folds, with respect to lowest value registered. Comparison among multiple groups was analyzed by one-way analysis of variance, followed by Bonferroni’s post hoc test.

**Figure 4:**
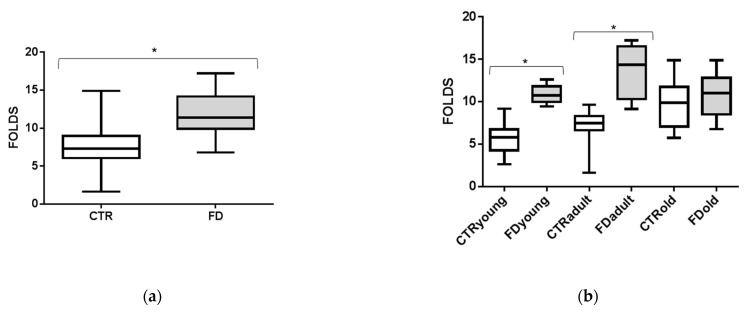
Comparisons of miR-126-3p levels among CTRs and FD patients. Box and Whiskers indicate median, minimum, and maximum levels of miR-126-3p in sEVs from plasma of all FD patients (30) and all CTRs (60) (**a**). Comparisons of miR-126-3p levels among CTRLs and FD patients subdivided into three age classes: young, adult, and old (**b**). CTs (cycle thresholds) resulting from qRT-PCR analysis were normalized with miR-30a; levels were calculated with 2-ΔCT method and expressed as folds with respect to lowest value registered. * *p* from *t*-test < 0.01.

MiR-126-3p levels were investigated in sEVs purified from FD patients’ plasma. FD patients were divided into young (18–46 years, *n* = 10) adult (58–70 years, *n* = 10) and old (75–82 years, *n* = 10) groups. We observed (Figure 3a) that sEVs-miR-126-3p levels were not age-related in FD patients (correlation coefficient R = 0.05). ANOVA, followed by Bonferroni’s post hoc test, showed significant differences in young FD patients, as compared to the adult group (Figure 3b). Furthermore, we compared sEVs-miR-126-3p levels between FD patients and CTRs matched for age. Overall, sEVs-miR-126-3p mean levels resulted in being significantly higher in FD patients (ratio FD vs. CTR: 1.6 folds), with respect to CTRs (Figure 4a). In particular, MiR-126-3p levels of FD patients were higher in young and adult groups (ratio young FD vs. young CTRs: 1.6 folds; ratio adult FD vs. adult CTRs: 2 folds), compared to CTRs (Figure 4b). Overall, in FD patients, sEVs-miR-126-3p levels were higher than the correspondent healthy age class and comparable to older healthy subjects. Therefore, we hypothesized that these data could explain the premature aging phenomena.

### 3.3. miR-126-3p Levels in HUVECs Undergoing Replicative Senescence

**Figure 5:**
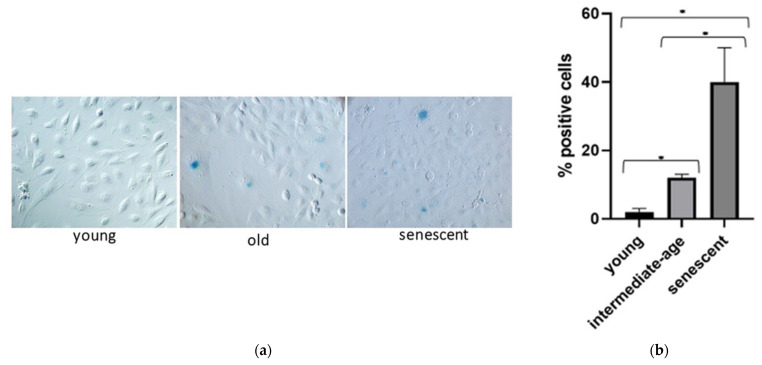
Human umbilical vein endothelial cells (HUVECs) senescence status. Senescence status was estimated by SA-β-Gal assay in different passage numbers; images are representative of three independent experiments (**a**). Replicative senescence. Data are expressed as % of SA-β-Gal positive cells and are the means ± SD (**b**). Comparison among multiple groups was analyzed by one-way analysis of variance, followed by Bonferroni’s post hoc test. * *p* < 0.01.

**Figure 6:**
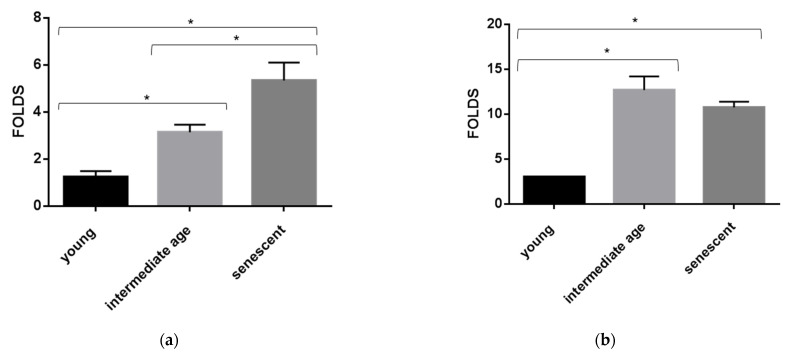
MiR-126a-3p levels variation in HUVECs undergoing senescence. Bar charts show sEVs (**a**) and intracellular (**b**) miR-126-3p levels in young, intermediate, and senescent HUVECs. Data were calculated by qRT-PCR and represent mean ± SD of three different experiments analyzed in triplicate. CTs (cycle thresholds) resulting from qRT-PCR analysis were normalized with miR-30a (EVs) and RNU6b (intracellular); levels were calculated with 2-ΔCT method and expressed as folds, with respect to lowest value registered. Comparison among multiple groups was analyzed by one-way analysis of variance, followed by Bonferroni’s post hoc test. * *p* < 0.01.

MiR-126-3p is an endothelial cell-specific miRNA, and endothelial cells senescence is known to be involved in aging process. We hypothesized that alterations of sEVs-miR-126-3p levels in aging could be linked to ECs senescence. Since it is difficult to study ECs’ senescence state in vivo, an in vitro model to investigate age-related changes of miR-126-3p in HUVECs and sEVs was used. In cultured HUVECs undergoing replicative senescence, intracellular and sEVs’ contained miR-126-3p levels were measured. Cell senescence was defined based on cumulative population doubling (CPD). We considered CPD > 56 for senescent cells, CPD < 32 for intermediate age cells, and CPD < 20 for young cells. Senescence status was estimated by SA-β-gal activity (Figure 5a) and measured as percentage of positive cells (40 ± 10 in senescent, 10 ± 4 in intermediate age and 2 ± 1 in young cells) (Figure 5b). Senescent and intermediate age ECs showed higher levels of miR-126-3p with respect to young cells (senescent vs. young ratio: 4 folds, and intermediate age vs. young ratio: 4.7 folds) (Figure 6b). MiR-126-3p was packaged in sEVs at progressively higher levels, with increasing senescence process (senescent vs young ratio: 4.5 folds and intermediate age vs. young ratio: 1.6 folds (Figure 6a). These results indicate that in vitro sEVs-miR-126-3p levels correlate with replicative senescent state and suggest that age-related variations of miR-126-3p in sEVs observed in vivo could be linked to endothelial cells’ senescence process.

### 3.4. ROS Production and Senescence in HUVECs Treated with Gb3 AND Lyso-Gb3 

**Figure 7:**
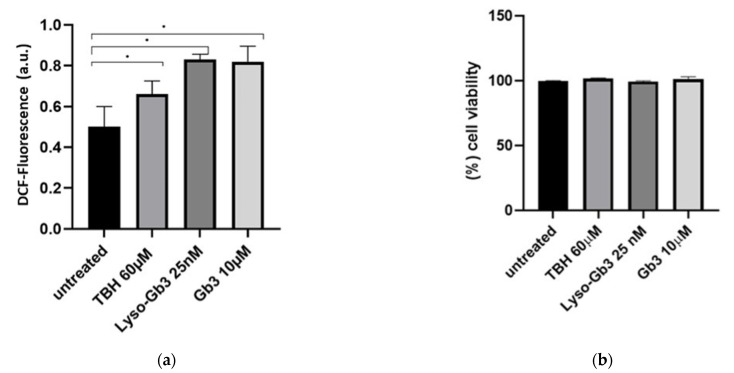
Lyso-Gb3 and Gb3 effect on HUVECs. Intracellular ROS levels and cell viability were assayed in HUVECs treated with TBH 60 µM, Lyso-Gb3 25 nM, and Gb3 10 µM. Intracellular ROS were measured as fluorescence intensity, using a DCFH-DA probe. The results, in arbitrary units (au), are presented as mean ± SD of three different experiments analyzed in triplicate (**a**). Cell viability was evaluated with MTS assay. The results presented as mean % of viable cells ± SD of three different experiments analyzed in triplicate (**b**). Comparison among multiple groups was analyzed by one-way analysis of variance, followed by Bonferroni’s post hoc test. * *p* < 0.01.

**Figure 8:**
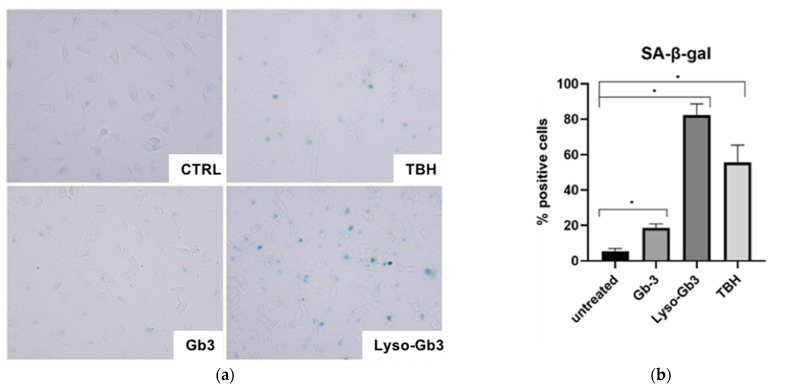
Lyso-Gb3 and GB3 effect on HUVECs senescence. Senescence status was estimated by SA-β-Gal assay in HUVECs treated with TBH 60 µM, Lyso-Gb3 25 nM, and Gb3 10 µM. Images are representative of ten random fields (**a**). Data are expressed as % of SA-b-Gal positive cells and are the means ± SD (**b**). Comparison among multiple groups was analyzed by one-way analysis of variance, followed by Bonferroni’s post hoc test. * *p* < 0.01.

**Figure 9:**
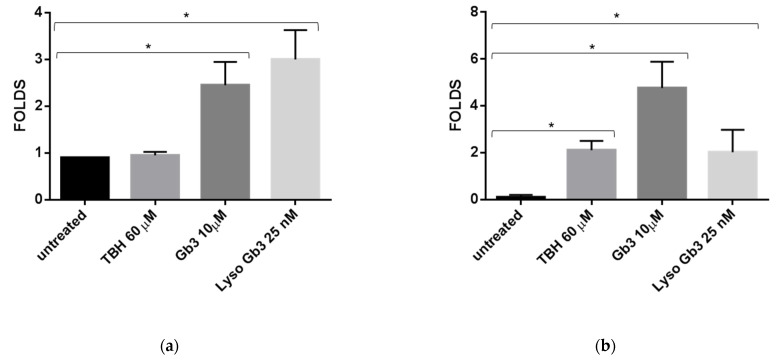
Variation in miR-126a-3p levels in HUVEC treated with TBH 60 µM, Lyso-Gb3 25 nM, and Gb3 10 µM. Bar charts show intracellular (**a**) and sEVs miR-126-3p levels (**b**). Data were calculated by qRT-PCR and represent mean ± SD of three different experiments analyzed in triplicate. CTs (cycle thresholds) resulting from qRT-PCR analysis were normalized with miR-30a (EVs) and RNU6b (intracellular); miR-126a-3p levels were calculated with 2-ΔCT method and expressed as folds, with respect to lowest value registered. Comparison among multiple groups was analyzed by one-way analysis of variance, followed by Bonferroni’s post hoc test. * *p* < 0.01.

In order to investigate on the roles of ECs senescence in FD, we evaluated the effects of Gb-3 and Lyso-Gb3, the most abundant substances accumulated in FD patients, into the cells and in extracellular space, respectively. Since the effects of Gb-3 and Lyso-Gb3 on ECs in vivo are extremely difficult to be investigated, we treated HUVECs with the two substrates to mimic FD pathogenic process. As oxidative stress is a major stimulus for senescence induction in ECs, we evaluated the effect of these substrates on oxygen reactive species (ROS) production. We analyzed ROS intracellular induction by using DCFH-DA assay in HUVECs treated with Gb3 and Lyso-Gb3 at concentrations comparable to those found in FD patients [5,13]. ROS levels induced by Gb3 and Lyso-Gb3 and Tert-Butyl Hydroperoxide (TBH), an exogenous inducer of oxidative stress, were comparable. In HUVECs treated with Gb3 and lyso-Gb3, ROS levels are similar to TBH treatment and higher than untreated cells. The fluorescence (evaluated as au: arbitrary units) of untreated cells is 0.5 au, and in HUVECs treated with TBH 60 µM, it is 0.7 au; in Lyso-Gb3 25 nM, it is 0.8 au; and in Gb3 10 µM, it is 0.8 au. (Figure 7a). These treatments did not cause cytotoxicity, as evaluated with MTS assay, in Figure 7b. In HUVECs, both Gb3 and Lyso-Gb3 treatments increased intracellular ROS production. Furthermore, since ROS can induce senescence in ECs, we evaluated HUVECs’ senescence state after treatment with the two substrates. Young HUVECs, with CPD < 20, were incubated with TBH (60 µM), Lyso-Gb3 (25 nM), and Gb3 (10 µM). SA-β-gal activity increased in treated HUVECs with respect of untreated cells. SA-β-gal activity is 5% in untreated cells, and in HUVECs treated with Gb3, it is 20%; in Lyso-Gb3, it is 80%; and in TBH, is 60%. SA-β-gal activity in HUVECs treated with Lyso-Gb3 was higher than TBH treatment (Figure 8a,b). Furthermore, we verified if HUVECs senescence induced by Gb3 and Lyso-Gb3 affected intracellular and sEVs miR-126-3p levels. We observed a significant increase of intracellular and sEVs miR-126-3p mean levels in HUVECs treated with Gb3 and Lyso-Gb3, as compared to untreated cells. Instead, the treatment of HUVECs with TBH increased EVs-miR-126-3p but did not affect intracellular miR-126-3p levels. In particular, we observed that EVs’ miR-126-3p is higher than control after treatment with TBH (4 ± 2 folds), Gb3 (15 ± 3 folds), and Lyso-Gb3 (6 ± 2 folds). The difference of treated HUVECs intracellular miR-126-3p levels were not significant with respect to controls (Figure 9a,b). Notably, we found that treatments of HUVECs with Gb3 and lyso-Gb3 caused oxidative stress and premature senescence, inducing miR-126-3p levels in sEVs and parental cells. These results identified a possible link between ECs senescence and the pathogenic mechanism of FD.

## 4. Discussion

In this work we explored the hypothesis that FD is a pathology associated with premature aging such as chronic kidney disease and chronic obstructive pulmonary disease [52]. Beyond the evidence that FD is associated with a significantly reduced life expectancy compared to the general population, other data support the link between FD and aging. Recent studies demonstrated the dysregulation of some aging hallmarks, like telomere attrition and DNA damage in FD patients or in experimental models [53]. To confirm these preliminary reports, we investigated on miRNAs as aging biomarker [54]; among the age-related miRNAs, we focused on miR-126-3p [55]. In our previous study, we found that miR-126-3p plasmatic levels were altered in FD patients [50]. The alterations of circulating miR-126-3p levels, associated with aging and aging related disease, has been observed in different studies, even if, with contrasting results [41,42]. Since senescent cells release more miRNAs contained in sEVs, with respect to normal cells, and miRNAs composition of sEVs is modified by senescence [56], we measured miR-126-3p age-related changes in sEVs. We found that miR-126-3p levels carried in sEVs increase progressively with age.

As miR-126-3p is endothelial cell-specific, we assumed the age-related miR-126-3p variations could be linked to ECs senescence. Based on the evidence that in vitro replicative senescence of ECs mimics the progressive age-related changes of endothelial function described in vivo, we used HUVECs undergoing replicative senescence as in vitro model [57]. We observed that HUVECs release EVs-miR-126-3p in progressively higher levels with increasing senescence process. Noteworthy, in FD patients miR-126-3p age-related trend was different with respect to healthy subjects. 

FD patients experience a progressive decline of glomerular filtration rate caused by renal injury. It is known that kidney function influence circulating miRNAs, plasmatic miR-126 levels have been found significantly and positively correlated with eGFR [58,59]. In this study, although eGFR decrease with age also in healthy subjects, FD patients showed, on average, lower levels of eGFR with respect CTR, as expected. This point could be considered a limit of the study, but surprisingly, our data showed higher levels of circulating miR-126 in FD patients compared to CTR groups. These observations would suggest that specific damages caused by Fabry disease, induce higher miR-126 plasmatic levels than kidney status.

The levels of sEVs-miR-126-3p in FD patients were higher than age matched CTRs. Our data indicate that FD patients are characterized by an accelerated ECs senescence and an altered age-related trend of miR-126-3p contained in sEVs. Moreover, in HUVECs, the treatments with Gb3 and Lyso-Gb3, simulating FD pathophysiology, induced senescence and increase of miR-126-3p release. Interesting, in HUVECs treated with Gb3 and Lyso-Gb3 also increased ROS production. These results indicate a potential link between ECs senescence and pathogenic mechanism of FD. Furthermore, according to free-radical theory, the highly reactive ROS are responsible for senescence [60]. When the redox balance is disturbed, the cells shift into an oxidative stress state, which subsequently leads to premature senescence [61,62]. Oxidative stress caused by GB-3 and Lyso-Gb3 is the probable cause of high levels of miR-126-3p in sEVs released from ECs. It was also reported that oxidative stress could affect extracellular miRNA profiles [63] and some transported miRNAs could exert cytotoxic or cytoprotective effects in recipient cells [63,64,65]. 

Moreover, the role of miR-126-3p targets suggest that increased levels of circulating miR-126-3p in FD patients could represent a protective mechanism against cell dysfunctions, consequent to premature senescence. One of miR-126-3p targets is SPRED-1 protein Spred-1 is an inhibitor of Ras/ERK signaling pathway, which is involved in the regulation of several cellular process including differentiation, survival, motility, and cell cycle. Delivery of MiR-126-3p via EVs could enhances cell survival through reduction of SPRED-1 and consequent activation of Ras/ERK/VEGF and PI3K/Akt/eNOS signaling pathways [38]. Another protective mechanism played by miR-126-3p could be linked to telomere homeostasis. A prominent molecular process underlying aging is the progressive shortening of telomeres which eventually triggers DNA damage response (DDR) [66]. DDR activation leads to increased levels of the transcription factor TP53 (p53), which is involved in DNA repair, cell cycle arrest, and apoptosis. Some evidence indicates that miR-126-3p controls the DDR by repressing ATM protein kinase activity in endothelial cells [67]. Interestingly, a rise in miR-126 abundance delays the senescence of human glomerular mesangial cells induced by high glucose. While high glucose leads to shortened telomeres, miR-126 upregulation is associated with extended telomeres and decreased expression of DDR and senescence markers TP53 and p21. Another study reported that deletion of miR-126a promotes hepatic aging in mice and induced age-associated telomere shortening [68]. Counteracting telomere shortening, miR-126-3p could limit senescence deleterious effects such as secretion of proinflammatory cytokines.

Our data suggest that sEVs collected from plasma of FD patients are released by senescent ECs and sEVs -miR-126-3p is useful as aging biomarker. Although the role of miR-126-3p in oxidative stress response and senescence was already known, in this study, for the first time, we described the link between senescence caused by oxidative stress, miR-126-3p release and lyso-Gb3 effects on endothelial cells. Future research could shed more light on the systemic nature of FD, clarifying the mechanisms directly related to substrate storage and investigating on new patterns potentially involved in FD. Further and more detailed studies are needed to discover new targets to prevent premature aging in FD as support to the conventional therapeutic strategies.

## Figures and Tables

**Table 1:** Characteristics of Fabry Disease (FD) patients and controls (CTRs).

	FD Patients 30 N° (%) (Mean) (Range)			CTRs 60 N° (%) (Mean) (Range)		
Males N°	12			35		
**Age**						
Young N° (range)	10 (18–46)			20 (24–44)		
Adult N° (range)	10 (58–70)			20 (53–68)		
Old N°(range)	10 (75–82)			20 (81–96)		
**GLA MUTATION**						
Missense N°	21			0		
Nonsense N°	3			0		
Frame shift N°	5			0		
Splicing site N°	1			0		
**AGal activity (range)**	(0.1–4.7)			ND		
**LysoGb3 (range)**	(4.8–43.8)			ND		
**Classical symptoms**						
Acroparesthesia N°	12			ND		
Cornea verticillata N°	5			ND		
Angiokeratoma N°	7			ND		
**Organ involvment**	**Young**	**Adult**	**old**	**Young**	**Adult**	**Old**
eGFR Male (mean)	118	93	45	133	97	56
eGFR Woman (mean)	91	63	26	99	88	36
LVH	0	3	2	0	0	0
Stroke/TIA	1	2	0	0	0	0
Myocardial infarct	0	0	0	0	0	0
**Cardiovascular risk factors**	**Young**	**Adult**	**old**	**Young**	**Adult**	**Old**
Diabete mellitus (%)	0	0	0	0	0	0
Hypertension (%)	0	40	10	0	21	17
Dislipidemia (%)	0	0	0	3	42	17
Smoking (%)	0	17	0	33	29	10
Obesity (%)	0	0	0	3	18	11

**Abbreviations:** GLA, alpha-galactosidase A; eGFR, estimated glomerular filtration rate; LVH, left ventricular hypertrophy; TIA, transient ischemic attack; ND, not determined. Enzyme activity was measured in nmol/mL/h; normal values > 3 nmol/mL/h. LysoGb3 is measured in nmol/L, normal values 0.08–1.13 nmol/L.

## References

[1] Flatt T. (2012). A new definition of aging?. Front. Genet..

[2] Fries J.F. (1980). Aging, natural death, and the compression of morbidity. N. Engl. J. Med..

[3] Burtner C.R., Kennedy B.K. (2010). Progeria syndromes and ageing: What is the connection?. Nat. Rev. Mol. Cell Biol..

[4] Ebert T., Pawelzik S.-C., Witasp A., Arefin S., Hobson S., Kublickiene K., Shiels P.G., Bäck M., Stenvinkel P. (2020). Inflammation and Premature Ageing in Chronic Kidney Disease. Toxins.

[5] Germain D.P. (2010). Fabry disease. Orphanet J. Rare Dis..

[6] Duro G., Zizzo C., Cammarata G., Burlina A., Burlina A., Polo G., Scalia S., Oliveri R., Sciarrino S., Francofonte D. (2018). Mutations in the GLA Gene and LysoGb3: Is It Really Anderson-Fabry Disease?. Int. J. Mol. Sci..

[7] Aerts J.M., Groener J.E., Kuiper S., Donker-Koopman W.E., Strijland A., Ottenhoff R., van Roomen C., Mirzaian M., Wijburg F.A., Linthorst G.E. (2008). Elevated globotriaosylsphingosine is a hallmark of Fabry disease. Proc. Natl. Acad. Sci. USA.

[8] Karetova D., Bultas J., Dostalova G., Palecek T., Kovarnik T., Golan L., Linhart A. (2010). Fabry disease—Vascular manifestations. Vasa.

[9] Biancini G.B., Moura D.J., Manini P.R., Faverzani J.L., Netto C.B.O., Deon M., Giugliani R., Saffi J., Vargas C.R. (2015). DNA damage in Fabry patients: An investigation of oxidative damage and repair. Mutat Res. Genet. Toxicol Environ. Mutagen..

[10] Bekaert S., De Meyer T., Van Oostveldt P. (2005). Telomere attrition as ageing biomarker. Anticancer Res..

[11] Kooman J.P., Stenvinkel P., Shiels P.G. (2020). Fabry Disease: A New Model of Premature Ageing?. Nephron.

[12] Kirkland J.L., Tchkonia T. (2017). Cellular Senescence: A Translational Perspective. EBioMedicine.

[13] Cokan Vujkovac A., Novaković S., Vujkovac B., Števanec M., Škerl P., Šabovič M. (2020). Aging in Fabry Disease: Role of Telomere Length, Telomerase Activity, and Kidney Disease. Nephron.

[14] Kumar S., Vijayan M., Bhatti J.S., Reddy P.H. (2017). MicroRNAs as Peripheral Biomarkers in Aging and Age-Related Diseases. Prog Mol. Biol. Transl. Sci..

[15] Bushati N., Cohen S.M. (2007). microRNA functions. Annu. Rev. Cell Dev. Biol..

[16] Crescitelli R., Lässer C., Szabó T.G., Kittel A., Eldh M., Dianzani I., Buzás E.I., Lötvall J. (2013). Distinct RNA profiles in subpopulations of extracellular vesicles: Apoptotic bodies, microvesicles and exosomes. J. Extracell Vesicles.

[17] Boon R.A., Vickers K.C. (2013). Intercellular transport of microRNAs. Arter. Thromb. Vasc. Biol..

[18] Théry C., Amigorena S., Raposo G., Clayton A. (2006). Isolation and characterization of exosomes from cell culture supernatants and biological fluids. Curr. Protoc. Cell Biol..

[19] Sohel M.H. (2016). Extracellular/Circulating MicroRNAs: Release Mechanisms, Functions and Challenges. Achiev. Life Sci..

[20] Pucci M., Reclusa Asiain P., Durendez Saez E., Jantus-Lewintre E., Malarani M., Khan S., Fontana S., Naing A., Passiglia F., Raez L.E. (2018). Extracellular Vesicles as miRNA Nano-Shuttles: Dual Role in Tumor Progression. Target Oncol..

[21] Fanale D., Taverna S., Russo A., Bazan V. (2018). Circular RNA in Exosomes. Adv. Exp. Med. Biol..

[22] Galvano A., Taverna S., Badalamenti G., Incorvaia L., Castiglia M., Barraco N., Passiglia F., Fulfaro F., Beretta G., Duro G. (2019). Detection of RAS mutations in circulating tumor DNA: A new weapon in an old war against colorectal cancer. A systematic review of literature and meta-analysis. Ther. Adv. Med. Oncol..

[23] Van Niel G., D’Angelo G., Raposo G. (2018). Shedding light on the cell biology of extracellular vesicles. Nat. Rev. Mol. Cell Biol..

[24] Raposo G., Stoorvogel W. (2013). Extracellular vesicles: Exosomes, microvesicles, and friends. J. Cell Biol..

[25] Thery C., Witwer K.W., Aikawa E., Alcaraz M.J., Anderson J.D., Andriantsitohaina R., Antoniou A., Arab T., Archer F., Atkin-Smith G.K. (2018). Minimal information for studies of extracellular vesicles 2018 (MISEV2018): A position statement of the International Society for Extracellular Vesicles and update of the MISEV2014 guidelines. J. Extracell Vesicles.

[26] Witwer K.W., Théry C. (2019). Extracellular vesicles or exosomes? On primacy, precision, and popularity influencing a choice of nomenclature. J. Extracell. Vesicles.

[27] Maas S.L.N., Breakefield X.O., Weaver A.M. (2017). Extracellular Vesicles: Unique Intercellular Delivery Vehicles. Trends Cell Biol..

[28] Terlecki-Zaniewicz L., Lämmermann I., Latreille J., Bobbili M.R., Pils V., Schosserer M., Weinmüllner R., Dellago H., Skalicky S., Pum D. (2018). Small extracellular vesicles and their miRNA cargo are anti-apoptotic members of the senescence-associated secretory phenotype. Aging.

[29] Olivieri F., Capri M., Bonafè M., Morsiani C., Jung H.J., Spazzafumo L., Viña J., Suh Y. (2017). Circulating miRNAs and miRNA shuttles as biomarkers: Perspective trajectories of healthy and unhealthy aging. Mech. Ageing Dev..

[30] Taverna S., Amodeo V., Saieva L., Russo A., Giallombardo M., De Leo G., Alessandro R. (2014). Exosomal shuttling of miR-126 in endothelial cells modulates adhesive and migratory abilities of chronic myelogenous leukemia cells. Mol. Cancer.

[31] El Andaloussi S., Mäger I., Breakefield X.O., Wood M.J.A. (2013). Extracellular vesicles: Biology and emerging therapeutic opportunities. Nat. Rev. Drug Discov..

[32] Taverna S., Fontana S., Monteleone F., Pucci M., Saieva L., De Caro V., Cardinale V.G., Giallombardo M., Vicario E., Rolfo C. (2016). Curcumin modulates chronic myelogenous leukemia exosomes composition and affects angiogenic phenotype via exosomal miR-21. Oncotarget.

[33] Cammarata G., Duro G., Di Chiara T., Lo Curto A., Taverna S., Candore G. (2019). Circulating miRNAs in Successful and Unsuccessful Aging. A mini-review. Curr. Pharm. Des..

[34] Venkat P., Cui C., Chopp M., Zacharek A., Wang F., Landschoot-Ward J., Shen Y., Chen J. (2019). MiR-126 Mediates Brain Endothelial Cell Exosome Treatment-Induced Neurorestorative Effects After Stroke in Type 2 Diabetes Mellitus Mice. Stroke.

[35] Chen C., Zhang L., Huang H., Liu S., Liang Y., Xu L., Li S., Cheng Y., Tang W. (2018). Serum miR-126-3p level is down-regulated in sepsis patients. Int. J. Clin. Exp. Pathol..

[36] Zernecke A., Bidzhekov K., Noels H., Shagdarsuren E., Gan L., Denecke B., Hristov M., Köppel T., Jahantigh M.N., Lutgens E. (2009). Delivery of microRNA-126 by apoptotic bodies induces CXCL12-dependent vascular protection. Sci. Signal..

[37] Harris T.A., Yamakuchi M., Ferlito M., Mendell J.T., Lowenstein C.J. (2008). MicroRNA-126 regulates endothelial expression of vascular cell adhesion molecule 1. Proc. Natl. Acad. Sci. USA.

[38] Meng S., Cao J.-T., Zhang B., Zhou Q., Shen C.-X., Wang C.-Q. (2012). Downregulation of microRNA-126 in endothelial progenitor cells from diabetes patients, impairs their functional properties, via target gene Spred-1. J. Mol. Cell Cardiol..

[39] Zhu N., Zhang D., Xie H., Zhou Z., Chen H., Hu T., Bai Y., Shen Y., Yuan W., Jing Q. (2011). Endothelial-specific intron-derived miR-126 is down-regulated in human breast cancer and targets both VEGFA and PIK3R2. Mol. Cell Biochem..

[40] Fish J.E., Santoro M.M., Morton S.U., Yu S., Yeh R.-F., Wythe J.D., Ivey K.N., Bruneau B.G., Stainier D.Y.R., Srivastava D. (2008). miR-126 regulates angiogenic signaling and vascular integrity. Dev. Cell..

[41] Wang S., Aurora A.B., Johnson B.A., Qi X., McAnally J., Hill J.A., Richardson J.A., Bassel-Duby R., Olson E.N. (2008). The endothelial-specific microRNA miR-126 governs vascular integrity and angiogenesis. Dev. Cell..

[42] Zampetaki A., Kiechl S., Drozdov I., Willeit P., Mayr U., Prokopi M., Mayr A., Weger S., Oberhollenzer F., Bonora E. (2010). Plasma microRNA profiling reveals loss of endothelial miR-126 and other microRNAs in type 2 diabetes. Circ. Res..

[43] Ravarotto V., Simioni F., Carraro G., Bertoldi G., Pagnin E., Calò L.A. (2018). Oxidative Stress and Cardiovascular-Renal Damage in Fabry Disease: Is There Room for a Pathophysiological Involvement?. J. Clin. Med..

[44] Metzinger-Le Meuth V., Metzinger L., Massy Z.A. (2014). miR-126 and miR-223 as biomarkers of vascular damage in the course of Chronic Kidney Disease. RNA Dis..

[45] Zou Q., Yang M., Yu M., Liu C. (2020). Influences of Regulation of miR-126 on Inflammation, Th17/Treg Subpopulation Differentiation, and Lymphocyte Apoptosis through Caspase Signaling Pathway in Sepsis. Inflammation.

[46] Shen J.-S., Meng X.-L., Moore D.F., Quirk J.M., Shayman J.A., Schiffmann R., Kaneski C.R. (2008). Globotriaosylceramide induces oxidative stress and up-regulates cell adhesion molecule expression in Fabry disease endothelial cells. Mol. Genet. Metab..

[47] Shu L., Vivekanandan-Giri A., Pennathur S., Smid B.E., Aerts J.M.F.G., Hollak C.E.M., Shayman J.A. (2014). Establishing 3-nitrotyrosine as a biomarker for the vasculopathy of Fabry disease. Kidney Int..

[48] Kaissarian N., Kang J., Shu L., Ferraz M.J., Aerts J.M., Shayman J.A. (2018). Dissociation of globotriaosylceramide and impaired endothelial function in α-galactosidase-A deficient EA.hy926 cells. Mol. Genet. Metab..

[49] Thum T., Tsikas D., Frölich J.C., Borlak J. (2003). Growth hormone induces eNOS expression and nitric oxide release in a cultured human endothelial cell line. FEBS Lett..

[50] Cammarata G., Scalia S., Colomba P., Zizzo C., Pisani A., Riccio E., Montalbano M., Alessandro R., Giordano A., Duro G. (2018). A pilot study of circulating microRNAs as potential biomarkers of Fabry disease. Oncotarget.

[51] Ying X., Wu Q., Wu X., Zhu Q., Wang X., Jiang L., Chen X., Wang X. (2016). Epithelial ovarian cancer-secreted exosomal miR-222-3p induces polarization of tumor-associated macrophages. Oncotarget.

[52] Kooman J.P., Shiels P.G., Stenvinkel P. (2015). Premature aging in chronic kidney disease and chronic obstructive pulmonary disease: Similarities and differences. Curr. Opin. Clin. Nutr. Metab. Care.

[53] Prasad K.N., Wu M., Bondy S.C. (2017). Telomere shortening during aging: Attenuation by antioxidants and anti-inflammatory agents. Mech Ageing Dev..

[54] Freedman J.E., Gerstein M., Mick E., Rozowsky J., Levy D., Kitchen R., Das S., Shah R., Danielson K., Beaulieu L. (2016). Diverse human extracellular RNAs are widely detected in human plasma. Nat. Commun..

[55] Olivieri F., Bonafè M., Spazzafumo L., Gobbi M., Prattichizzo F., Recchioni R., Marcheselli F., La Sala L., Galeazzi R., Rippo M.R. (2014). Age- and glycemia-related miR-126-3p levels in plasma and endothelial cells. Aging.

[56] Wang J., Chen S., Ma X., Cheng C., Xiao X., Chen J., Liu S., Zhao B., Chen Y. (2013). Effects of endothelial progenitor cell-derived microvesicles on hypoxia/reoxygenation-induced endothelial dysfunction and apoptosis. Oxid. Med. Cell Longev..

[57] Boisen L., Drasbek K.R., Pedersen A.S., Kristensen P. (2010). Evaluation of endothelial cell culture as a model system of vascular ageing. Exp. Gerontol..

[58] Ortiz A., Oliveira J.P., Waldek S., Warnock D.G., Cianciaruso B., Wanner C. (2008). Nephropathy in males and females with Fabry disease: Cross-sectional description of patients before treatment with enzyme replacement therapy. Nephrol. Dial. Transpl..

[59] Fourdinier O., Schepers E., Metzinger-Le Meuth V., Glorieux G., Liabeuf S., Verbeke F., Vanholder R., Brigant B., Pletinck A., Diouf M. (2019). Serum levels of miR-126 and miR-223 and outcomes in chronic kidney disease patients. Sci. Rep..

[60] Beckman K.B., Ames B.N. (1998). The free radical theory of aging matures. Physiol. Rev..

[61] Bu H., Wedel S., Cavinato M., Jansen-Dürr P. (2017). MicroRNA Regulation of Oxidative Stress-Induced Cellular Senescence. Oxid. Med. Cell Longev..

[62] Puca A.A., Carrizzo A., Villa F., Ferrario A., Casaburo M., Maciąg A., Vecchione C. (2013). Vascular ageing: The role of oxidative stress. Int. J. Biochem. Cell Biol..

[63] Dluzen D.F., Noren Hooten N., Evans M.K. (2017). Extracellular RNA in aging. Wiley Interdiscip. Rev. RNA.

[64] Matsuzaki J., Ochiya T. (2018). Extracellular microRNAs and oxidative stress in liver injury: A systematic mini review. J. Clin. Biochem. Nutr..

[65] Harrell C.R., Jovicic N., Djonov V., Arsenijevic N., Volarevic V. (2019). Mesenchymal Stem Cell-Derived Exosomes and Other Extracellular Vesicles as New Remedies in the Therapy of Inflammatory Diseases. Cells.

[66] Paschalaki K.E., Zampetaki A., Baker J.R., Birrell M.A., Starke R.D., Belvisi M.G., Barnes P.J. (2018). Downregulation of microRNA-126 augments DNA damage response in cigarette smokers and patients with chronic obstructive pulmonary disease. Am. J. Respir. Crit. Care Med..

[67] Cao D.W., Jiang C.M., Wan C., Zhang M., Zhang Q.Y., Zhao M., Han X. (2018). Upregulation of MiR-126 delays the senescence of human glomerular mesangial cells induced by high glucose via telomere-p53-p21-Rb signaling pathway. Curr. Med. Sci..

[68] Yan Y., Qin D., Hu B., Zhang C., Liu S., Wu D., Zhang L. (2019). Deletion of miR-126a promotes hepatic aging and inflammation in a mouse model of cholestasis. Mol. Ther. Nucleic Acids.

